# MiR-96-5p alleviates inflammatory responses by targeting NAMPT and regulating the NF-κB pathway in neonatal sepsis

**DOI:** 10.1042/BSR20201267

**Published:** 2020-07-03

**Authors:** Xueli Chen, Ying Chen, Li Dai, Na Wang

**Affiliations:** 1 Department of Pediatrics, Maternal and Child Health Hospital of Hubei Province (Women and Children’s Hospital of Hubei Province), Wuhan, Hubei, China; 2 Department of Pediatrics, Wuhan No. 1 Hospital, Wuhan, Hubei, China

**Keywords:** inflammatory, miR-96-5p, NAMPT, neonatal sepsis, NF-κB pathway

## Abstract

Neonatal septicemia is a serious infectious disease in the neonatal period. MicroRNAs (miRNAs) have been reported to participate in the inflammatory responses in neonatal sepsis. The aim of the present study was to explore the effects and molecular mechanism of miR-96-5p on regulating the inflammatory responses in neonatal sepsis. MiR-96-5p was low expressed while nicotinamide phosphoribosyltransferase (NAMPT) was high expressed in the serum of neonatal septicemia patients. The expression of miR-96-5p was decreased in LPS-induced inflammatory responses. Besides, miR-95-5p relieved LPS-induced inflammatory responses in RAW264.7 cells. NAMPT was demonstrated as a potential target of miR-96-5p, and knockdown of NAMPT reduced inflammatory in RAW264.7 cells stimulated with LPS. Moreover, overexpression of NAMPT reversed the effects of miR-96-5p on LPS-induced inflammatory responses. In addition, miR-96-5p inhibited nuclear factor (NF)-κB signaling pathway in RAW264.7 cells stimulated with LPS. MiR-96-5p alleviated inflammatory responses via targeting NAMPT and inhibiting NF-κB pathway in neonatal sepsis.

## Introduction

Neonatal sepsis is a common disease in newborn infants and has high morbidity and mortality [1]. It is the third leading cause of neonatal death and accounts for approximately 25% of neonatal mortality [2]. Sepsis is caused by disorder reactions in response to infection, leads to severe inflammatory responses and immune disorder [3]. In spite of tremendous efforts and advances in neonatology, early diagnosis and initiation of treatment of neonatal sepsis are still challenges because of nonspecific signs and symptoms, no perfect early diagnostic marker [4]. Therefore, it is urgent to search for new targets for early diagnosis and therapeutic of neonatal sepsis.

Increasing evidences indicate that MicroRNAs (miRNAs) participate in the regulation of immune response. MiRNAs are a kind of short noncoding RNAs containing 19–22 nucleotides. It suppresses mRNA expression by combining with the 3′-untranslated region (UTR) of target genes and resulting degradation or transcriptional inhibition of target mRNA [5,6]. Certain miRNAs have been reported to play roles in inflammatory responses. For example, microRNA-300 promotes inflammatory responses by targeting nicotinamide phosphoribosyltransferase (NAMPT) and activation of AMPK/mTOR pathway in neonatal sepsis [7]; miRNA-138 accelerates inflammatory responses via binding its target SIRT1 and activating the NF-κB and AKT pathways [8]; in contrast, miR-15a/16 restrains inflammatory responses induced by LPS [9]. Previous studies have shown that miR-96-5p has a low expression in leukocytes of neonatal septicemia patients [10]. Additionally, miR-96-5p regulates spinal cord injury through the NF-κB pathway [11]. However, it is unclear whether miR-96-5p plays a role in neonatal septicemia.

NAMPT also named as Pre-B-cell colony-enhancing factor (PBEF) or visfatin, which is a limiting enzyme in the nicotinamide adenine dinucleotide (NAD^+^) salvage biosynthetic pathway. It has been proved that NAMPT served as an inflammatory adipocytokine to involve in cell metabolism, inflammation and immune modulation [12,13]. A previous study indicated that NAMPT expression was elevated in neonatal sepsis, and was associated with inflammatory responses, suggesting that NAMPT was a vital regulator in inflammatory reactions [7].

In the present study, we first demonstrated that miR-96-5p participated in inflammatory responses through suppressing its target gene NAMPT and NF-κB pathway in neonatal sepsis, which may provide a theoretical basis for research on diagnosis and treatment of neonatal sepsis.

## Materials and methods

### Samples collection

After approved by Ethics Committee of Maternal and Child Health Hospital of Hubei Province (Women and Children’s Hospital of Hubei Province). The blood samples from 30 neonatal sepsis patients and 24 respiratory infection/pneumonia patients (control group) were obtained before the patients enrolled from Maternal and Child Health Hospital of Hubei Province (Women and Children’s Hospital of Hubei Province) that had not undergone any other therapy. Informed consent for all samples was written by patients’ families.

### Cell culture and treatment

The RAW264.7 murine macrophage cell line and HEK293T cells were purchased from the Cell Bank of the Chinese Academy of Sciences (Shanghai, China). All cell lines were cultured in Roswell Park Memorial Institute (RPMI) 1640 medium (Gibco, Carlsbad, CA, U.S.A.) with additional 10% fetal bovine serum (FBS, Gibco) at the condition of 37°C and 5% CO_2_ in a humidified atmosphere. LPS was used to stimulate macrophages cell treated with 0–2 μg/ml of LPS for 12 h or with 1 μg/ml of LPS for 0–48 h.

### Cell transfection

MiR-96-5p mimics (miR-96-5p), anti-miR-96-5p, small interfering RNA against NAMPT (si-NAMPT) and corresponding negative controls (NC) were designed and synthesized from Ribobio (Guangzhou, China). Full length of NAMPT cDNA was cloned and inserted in pcDNA3.1 vector (Invitrogen, Carlsbad, CA, U.S.A.) for the overexpression of NAMPT. RAW264.7 cells were inoculated on six-well plates; transfection was performed after the confluence of the cells was ∼70% by Lipofectamine 2000 reagent (Invitrogen) following the manufacturer’s instructions.

### RNA extraction and reverse transcription-quantitative PCR (qRT-PCR)

Total RNA was extracted from serum and cells by Trizol Reagent following the manufacturer’s protocol. Subsequently, cDNA was obtained by using TIANScript RT Kit (Tiangen Biotech, Beijing, China). Then, the expression levels were detected by qRT-PCR using an SYBR Green QuantiTect RTPCR Kit (Roche, South San Francisco, CA, U.S.A.) following the manufacturer’s instructions. U6 and GAPDH were employed to normalize miRNA and mRNA, respectively. Data were calculated by the 2^−∆∆Ct^ method. The primers for the tested genes were presented as follows: GAPDH-Forward: GTGTTCCTACCCCCAATGTG, GAPDH-Reverse: CATCGAAGGTGGAAGAGTGG; TNF-α-Forward: CGTCAGCCGATTTGCTATCT, TNF-α-Reverse: CTTGGGCAGATTGACCTCAG; IL-6-Forward GGGACTGATGCTGGTGACAA, IL-6-Reverse: TCCACGATTTCCCAGAGAACA; NAMPT-Forward: TACAGTGGCCACAAATTCCA, NAMPT-Reverse: CAATTCCCGCCACAGTATCT; U6-Forward: GCTTCGGCAGCACATATACTAAAAT, U6-Reverse: CGCTTCACGAATTTGCGTGTCAT; MiR-96-5p-Forward: ACGATGCACCTGTACGATCA, MiR-96-5p-Reverse: TCTTTCAACACGCAGGACAG.

### Western blot analysis

The proteins from blood samples and cells were extracted using RIPA lysis buffer following standard protocols, and protein concentrations were detected using protein assay kit (Bio-Rad Laboratories, Tokyo, Japan). About 30 μg proteins of each sample were separated by SDS-PAGE and then transferred to a PVDF membrane. After blocking for 1 h with 5% skim milk (dissolved in TBST buffer containing 0.1% Tween 20), the membrane was incubated with primary antibodies at 4°C overnight. After that, the membrane was washed with PBS three times and incubated with secondary antibodies at 37°C for 1 h. Blots were detected by enhanced chemiluminescence. The primary antibodies were rabbit antibodies, including anti-NAMPT, anti-p65, anti-p-p65, anti-IKKβ, anti-p-IKKα/β and anti-GAPDH as control (Abcam, Cambridge, U.K.), and horseradish peroxidase (HRP)-conjugated secondary antibodies were obtained from Abcam.

### Enzyme-linked immunosorbent assay (ELISA)

Anti-mouse TNF-α and IL-6 antibodies and biotinylated secondary antibodies were obtained from Abcam. The ELISA assay was carried out by using enzyme-linked immunosorbent assay (ELISA) kits (Abcam) according to the manufacturer’s protocol.

### Dual-luciferase reporter assay

Targetscan software has predicted the binding sequences between NAMPT and miR-96-5p. In order to confirm the relationship between NAMPT and miR-96-5p, dual luciferase reporter assay was performed as previously described [14]. The fragment of NAMPT 3′-UTR (NAMPT-WT) containing the predicted binding sites of miR-96-5p and its mutant (NAMPT-MUT) sequences were cloned into the downstream of the luciferase gene in pGL-control reporter plasmid vector (Promega, Madison, WI, U.S.A.), separately. The reporter vectors containing NAMPT-WT or NAMPT-MUT with miR-95-5p or miR-NC were co-transfected into HEK293T and RAW264.7 cells using Lipofectamine 2000. The luciferase activities of renin and firefly were measured using a dual-luciferase reporter assay system (Promega) after 36 h.

### Statistical analysis

At least three independent experiments were employed for all assays. Values were shown as the mean values ± standard deviation (SD). Differences were assessed through Student’s *t*-test or one-way analysis of variance by using SPSS 22.0 software. *P*<0.05 was deemed to be statistically significant.

## Results

### MiR-96-5p was down-regulated while NAMPT was up-regulated in neonatal sepsis

**Figure 1:**
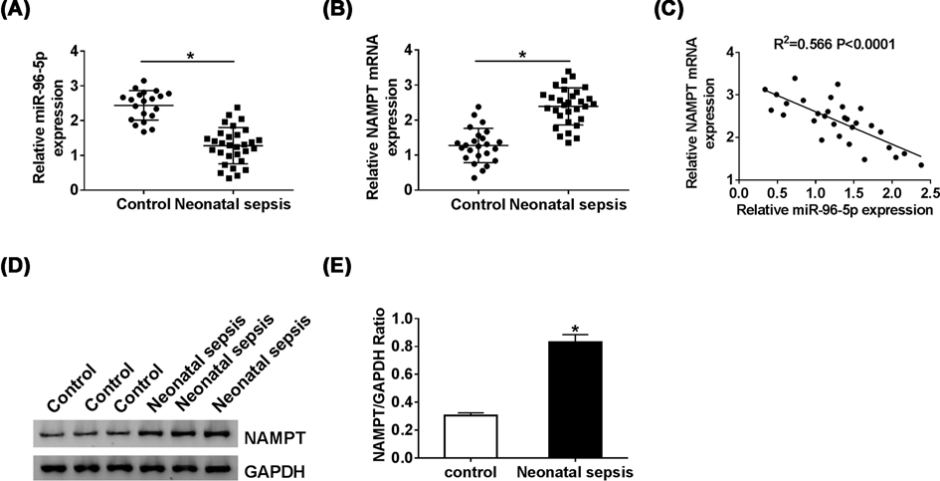
The expression levels of miR-96-5p and NAMPT in serum of neonatal sepsis patients (**A**) miR-96-5p level in serum of neonatal sepsis patients was detected by qRT-PCR. (**B**) the mRNA level of NAMPT in serum of neonatal sepsis patients was detected by qRT-PCR. (**C**) the negative correlation miR-96-5p and NAMPT mRNA in serum of neonatal sepsis patients was showed. (**D** and** E**) Western blot analysis was performed to measure the protein level of NAMPT in serum of neonatal sepsis patients; *P<0.05.

The expression levels of miR-96-5p and NAMPT were first detected in neonatal sepsis and control serum by qRT-PCR, and the result shown that miR-96-5p level was significantly decreased in the serum of neonatal sepsis compared with the control (Figure 1A). In contrast, the expression of NAMPT was up-regulated serum of neonatal sepsis patients (Figure 1B). Then, we analyzed the relationship between miR-96-5p and NAMPT, and the data indicated that the expression of miR-96-5p was negatively correlated with NAMPT in neonatal sepsis (Figure 1C). In addition, the NAMPT protein level was detected using Western blot, and the result showed that NAMPT expression was increased in neonatal sepsis (Figure 1D,E), which was consistent with the qRT-PCR result.

### MiR-96-5p negatively regulated LPS-induced inflammatory responses in RAW264.7 cells

**Figure 2:**
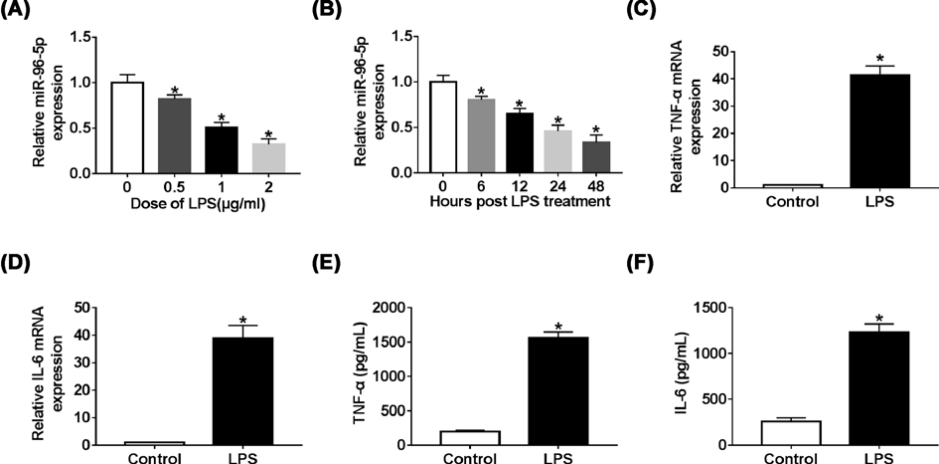
The expression level of miR-96-5p was suppressed and inflammatory response was induced by LPS (**A** and** B**) The level of miR-96-5p was measured in RAW264.7 cells treated with LPS at different dose and treatment hours. (**C** and** D**) The mRNA levels of TNF-α and IL-6 in RAW264.7 cells treated with LPS were detected by qRT-PCR. (**E** and** F**) The levels of TNF-α and IL-6 in RAW264.7 cells treated with LPS were detected by ELISA; *P<0.05.

Previous studies have demonstrated that LPS can stimulate macrophages and lead to inflammatory responses [15,16]. To confirm whether miR-96-5p was down-regulated in inflammatory responses, we measured the expression level of miR-96-5p in RAW264.7 cells stimulated with LPS of different dose or processing time. The data revealed that the expression of miR-96-5p was down-regulated by LPS in dose-dependent or time-dependent manners (Figure 2A,B). Additionally, the levels of inflammatory cytokines TNF-α and IL-6 were also detected to assess inflammatory responses induced by LPS, the qRT-PCR and ELISA results showed that levels of TNF-α and IL-6 were significantly increased in RAW264.7 cells stimulated with LPS (Figure 2C–F). These results suggested that miR-96-5p played a potential role in the regulation of LPS-induced inflammatory responses.

**Figure 3:**
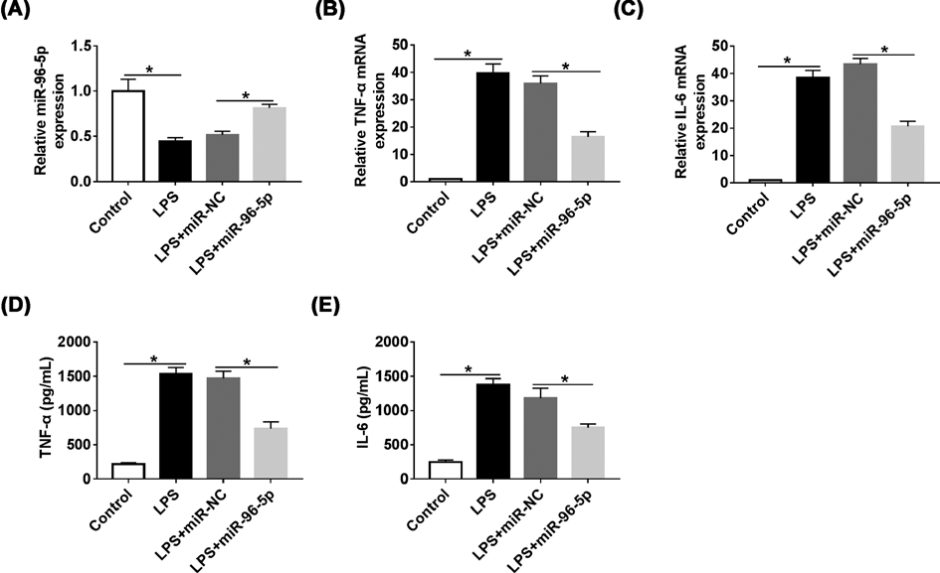
MiR-96-5p inhibited inflammatory response in RAW264.7 cells stimulated with LPS (**A**) The expression levels of miR-96-5p in RAW264.7 cells treated with LPS or LPS + miR-96-5p mimic were detected by qRT-PCR. (**B** and** C**) The mRNA levels of TNF-α and IL-6 in RAW264.7 cells treated with LPS or LPS + miR-96-5p mimic were detected by qRT-PCR. (**D** and** E**) The levels of TNF-α and IL-6 in RAW264.7 cells treated with LPS or LPS + miR-96-5p mimic were detected by ELISA; *P<0.05.

To investigate the role of miR-95-5p in inflammatory responses, miR-96-5p mimic or control miR-NC was transfected into RAW264.7 cells. As shown in Figure 3A, miR-96-5p expression was down-regulated by LPS, which was reversed when transfected with miR-96-5p. In addition, overexpression of miR-96-5p significantly decreased TNF-α and IL-6 levels in LPS-induced inflammatory responses in RAW264.7 cells (Figure 3B–E). Furthermore, to make these results more convincing, we also collected monocytes from the blood of neonatal sepsis patients. The qRT-PCR results indicated that overexpression of miR-96-5p down-regulated LPS-induced TNF-α and IL-6 expressions in monocytes (Supplementary Figure S1), which indicted that miR-96-5p relieved LPS-induced inflammatory response.

### NAMPT was directly regulated by miR-96-5p

**Figure 4:**
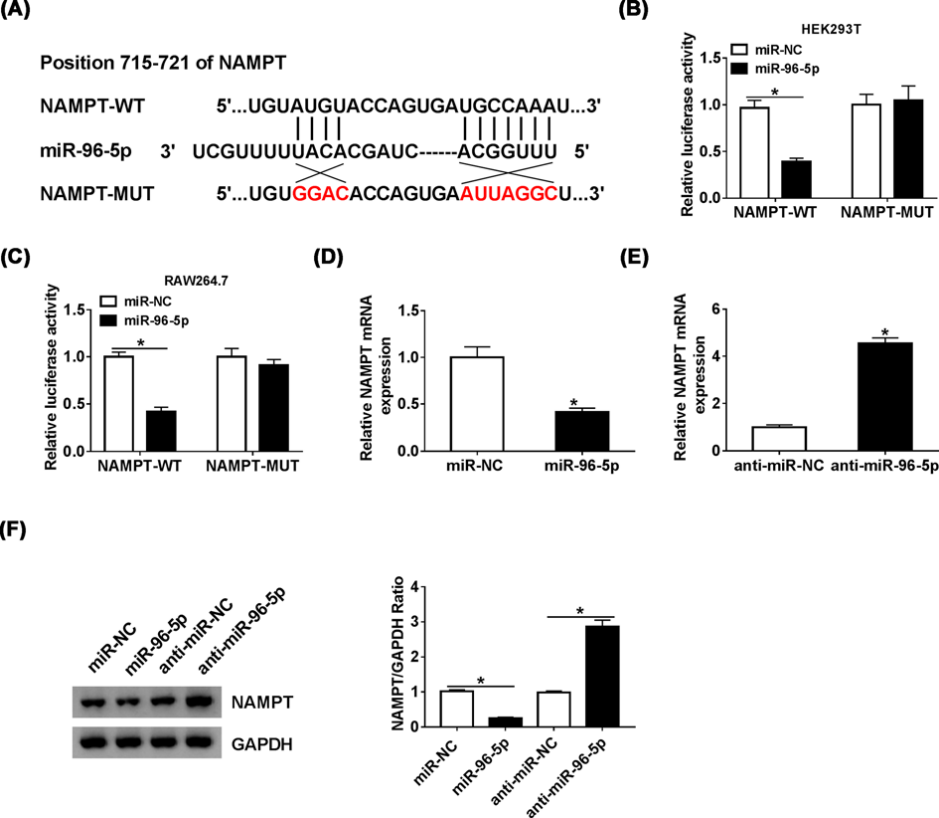
MiR-96-5p regulated NAMPT by directly binding NAMPT (**A**) The binding sites of miR-96-5p on NAMPT 3′-UTR were predicted by Targetscan software. (**B** and** C**) The luciferase activity was detected in HEK293T and RAW264.7 cells co-transfected with miR-96-5p and NAMPT-WT or NAMPT-MUT. (**D** and **E**) The mRNA levels of NAMPT in RAW264.7 cells transfected with miR-96-5p mimic or anti-miR-96-5p were detected by qRT-PCR. (**F**) The protein levels of NAMPT in RAW264.7 cells transfected with miR-96-5p mimic or anti-miR-96-5p were detected by Western blot analysis; *P<0.05.

To investigate the molecule mechanism by which miR-95-5p regulating inflammatory response, Targetscan software was used to predict the downstream target of miR-96-5p and identified NAMPT as a potential target, moreover, the binding site between miR-96-5p and NAMPT was also predicted (Figure 4A). Subsequently, dual luciferase reporter assay showed that the luciferase activity was strongly decreased by miR-96-5p mimics in both HEK239T and RAW264.7 cells co-transfected with NAMPT-WT luciferase reporter plasmid, but the luciferase activity had no change when NAMPT was mutational (Figure 4B,C) which confirmed that NAMPT was a direct target of miR-96-5p. Besides, we detected the expression level of NAMPT in RAW264.7 cells transfected with miR-96-5p or anti-miR-96-5p, qRT-PCR and Western blot results revealed NAMPT expression was decreased in cells transfected with miR-96-5p, while knockdown of miR-96-5p enhanced NAMPT level (Figure 4D–F).

### Knockdown of NAMPT relieved LPS-induced inflammatory response

**Figure 5:**
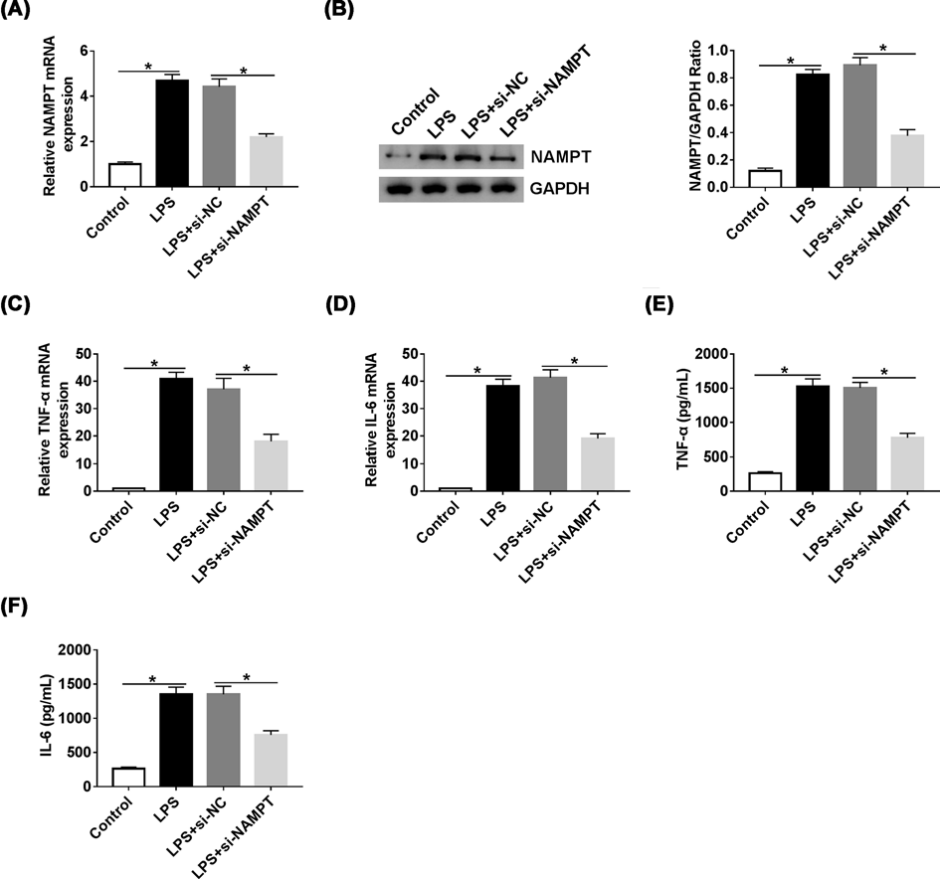
Knockdown of NAMPT relieved LPS-induced inflammatory response (**A** and** B**) The level of NAMPT in RAW264.7 cells treated with LPS or LPS + si-NAMPT was detected by qRT-PCR and Western blot. (**C** and **D**) The mRNA levels of TNF-α and IL-6 in RAW264.7 cells treated with LPS or LPS + si-NAMPT were detected by qRT-PCR. (**E** and** F**) The levels of TNF-α and IL-6 in RAW264.7 cells treated with LPS or LPS + si-NAMPT were detected by ELISA; *P<0.05.

To study the role of NAMPT in inflammatory response, si-NAMPT was transfected in RAW264.7 cells. The expression of NAMPT was up-regulated by LPS and reversed after transfected with si-NAMPT (Figure 5A,B). In addition, TNF-α and IL-6 were observably induced by LPS. However, knockdown of NAMPT decreased the expression of TNF-α and IL-6 (Figure 5C–F). These data suggested that knockdown of NAMPT suppressed inflammatory response.

### NAMPT overexpression reversed effects of miR-96-5p on LPS-induced inflammatory response

**Figure 6:**
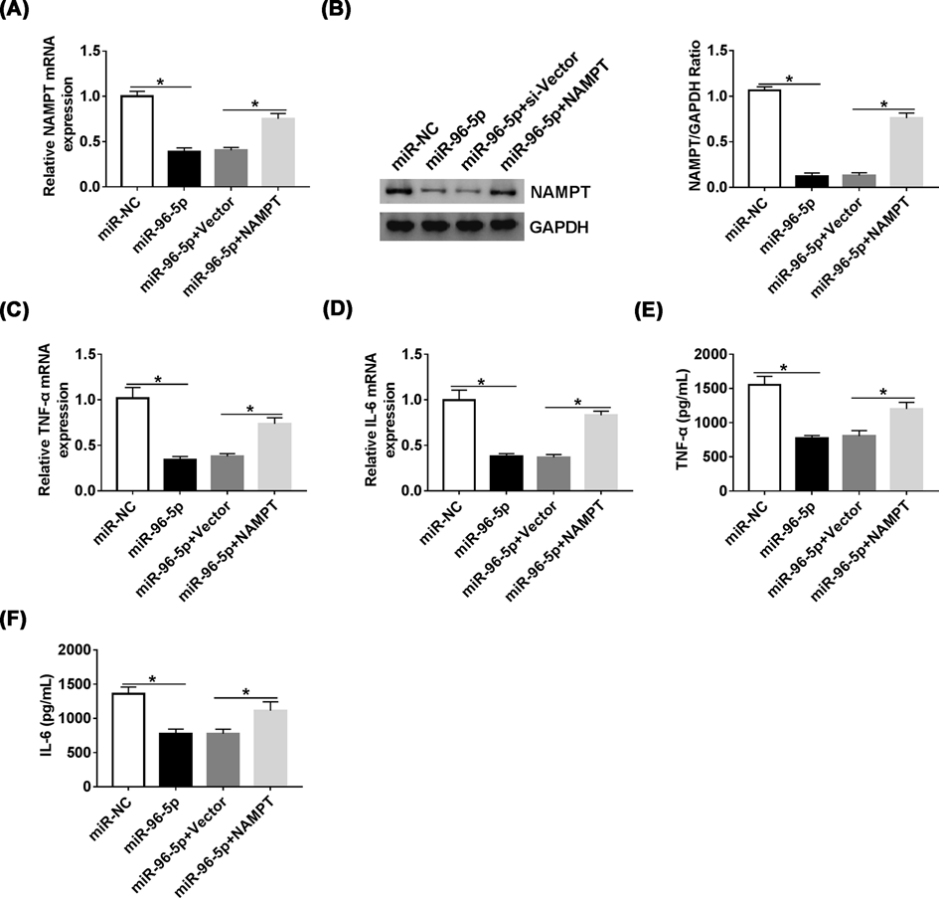
NAMPT overexpression reversed effects of miR-96-5p on LPS-induced inflammatory response (**A** and** B**) The level of NAMPT in RAW264.7 cells transfected with miR-96-5p mimic or miR-96-5p mimic + NAMPT was detected by qRT-PCR and Western blot. (**C** and **D**) The mRNA levels of TNF-α and IL-6 in RAW264.7 cells transfected with miR-96-5p or miR-96-5p + NAMPT were detected by qRT-PCR. (**E** and **F**) The levels of TNF-αand IL-6 in RAW264.7 cells transfected with miR-96-5p or miR-96-5p + NAMPT were detected by ELISA; *P<0.05.

To further investigate the interaction between miR-96-5p and NAMPT, the mRNA and protein levels of NAMPT were measured in RAW264.7 cells transfected miR-96-5p or co-transfected miR-96-5p and NAMPT. Overexpression of miR-96-5p decreased the level of NAMPT and overexpression of NAMPT could reverse the decreased expression (Figure 6A,B). Furthermore, we evaluated the inflammatory response through the expression of TNF-α and IL-6, the results revealed that miR-96-5p inhibits inflammatory and NAMPT overexpression reversed effects of miR-96-5p on LPS-induced inflammatory response (Figure 6C–F), which indicating that miR-96-5p regulated LPS-induced inflammatory response in RAW264.7 cells through suppressing NAMPT.

### MiR-96-5p inhibited NF-κB pathway in RAW264.7 cells stimulated with LPS

**Figure 7:**
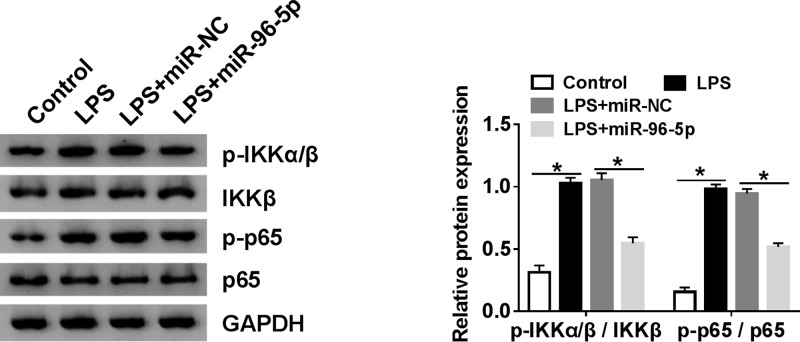
miR-96-5p inhibited NF-κB pathway in RAW264.7 cells stimulated with LPS The levels of p65, p-p65, IKKβ and p-IKKα/β in RAW264.7 cells treated with LPS or LPS + miR-96-5p were detected by Western blot. The ratios of p-IKKα/β/IKKβ and p-p65/p65 were analyzed; *P<0.05.

To investigate whether miR-96-5p involved in NF-κB pathway in LPS-induced inflammatory response, protein levels of p-IKKα/β, IKKβ, p-p65 and p65 were determined by using Western blot analysis. The ratio of p-IKKα/β/IKKβ and p-p65/p65 was calculated and acted as indicators of the activity of NF-κB pathway. The data indicated NF-κB pathway was activated by LPS, inversely, overexpression of miR-96-5p inhibited NF-κB pathway (Figure 7).

## Discussion

Sepsis is a complex syndrome caused by infection and is featured on a systemic inflammatory response. Because the adaptive immune system of newborns has not fully developed, the incidence of sepsis is extremely high in newborns [17]. Although the definition of sepsis was clear, the diagnosis of neonatal sepsis relies on the subjective interpretation of each case due to nonspecific signs and symptoms and no perfect early diagnostic marker [18]. So improving diagnostic and prognostic accuracy in neonatal sepsis would contribute to the therapy and reduce mortality of neonatal sepsis.

Inflammatory cytokines are critical factors in the inflammatory response. When pathogens infect the body, macrophages and dendritic cells can produce cytokines to resist the invasion of pathogens. To some extent, the levels of immune factors can be used to assess the immune response. TNF-α and IL-6 are pro-inflammatory cytokines, which involved in various inflammatory responses [19,20]. In the present study, the expression levels of TNF-α and IL-6 were enhanced by LPS, suggesting that the inflammatory response was stimulated by LPS, which was in agreement with a previous study [21].

The inflammatory response has potentially destructiveness, which makes it significant to regulate the inflammatory response finely. Studies of miRNAs suggest that they function as potential key regulators of the inflammatory response in many diseases, including neonatal sepsis [22–24]. MiR-15a/16 are increased in neonatal sepsis patients and can serve as predictors of neonatal sepsis [9]. A recent study has reported that the aberrant expression of miR-132 and miR-223 was connected with the inflammatory response in neonatal sepsis [22]. MiR-96-5p has been shown to be low expressed in neonatal sepsis patients [10]. However, the functional mechanism of miR-96-5p in neonatal sepsis is little known. In the present study, we also observed a decreased expression of miR-96-5p in the serum of neonatal sepsis patients. Moreover, the mRNA level of miR-96-5p was down-regulated by LPS in dose-dependent and treatment time-dependent manners. Subsequently, a gain of function method was used to investigate the role of miR-96-5p, and the result indicated that miR-96-5p overexpression inhibited the inflammatory response in RAW264.7 cells stimulated with LPS.

Generally, miRNA suppressed mRNA expression by the direct sponging target gene. Bioinformatics analysis was performed and showed that NAMPT contained binding sites for miR-96-5p. Then, dual-luciferase reporter assay proved that NAMPT was a target of miR-96-5p. NAMPT has been indicated as a vital regulator in inflammatory responses [25–27]. Inhibiting endogenous NAMPT alleviates diabetic nephropathy inflammatory-fibrosis by regulating NF-κB p65 and Sirt1 pathways [28]. A previous study demonstrated the pro-inflammatory effects of NAMPT involved the regulation of the insulin signaling pathway [29]. In the present study, we found NAMPT expression was increased in the serum of neonatal sepsis patients and induced by LPS in RAW264.7 cells. In addition, knockdown of NAMPT inhibited LPS-induced inflammatory response, which was consistent with previous studies [7,30].

The nuclear factor (NF)-κB signaling has been reported to be an important factor involved in innate immunity through regulating the transcription of pro-inflammatory genes [31,32]. The activation of the NF-κB signaling pathway mainly relies on some inflammatory signals, such as LPS, TNF-α, IL-1β, reactive oxygen species and so on [33–35]. Usually, NF-κB was inactivated by binding IκB proteins in the cytoplasm [36]. Free NF-κB will be released after phosphorylation and degradation of IκB, and then, NF-κB move to the nucleus, thereby bind to the target gene and promote its transcription [37]. Our study showed that overexpression of miR-96-5p inhibited NF-κB signaling pathway in RAW264.7 cells stimulated with LPS. Furthermore, previous study verified that TAK1 as an upstream kinase affected the NF-κB signaling pathway [38]. We also found that miR-96-5p could inhibit the phosphorylation of TAK1 in RAW264.7 cells stimulated with LPS (Supplementary Figure S2). These data indicated that miR-96-5p suppressed NF-κB pathway by weakening the activation of TAK1. In addition, a previous study indicated NAMPT also played a role in NF-κB signaling pathway [28], whether miR-96-5p inhibited NF-κB via targeting NAMPT and the underlying mechanisms remain to be further investigated.

In summary, our study shown that the decreased miR-96-5p expression was associated with elevated level of NAMPT and inflammatory response. The results demonstrated that miR-96-5p alleviated LPS-induced inflammatory response via targeting NAMPT and regulation of NF-κB signaling pathway. Understanding the effects and the functional mechanisms of miR-96-5p in inflammatory response might provide a theoretical basis for the diagnosis and therapy in neonatal sepsis.

## Supplementary Material

Supplementary Figures S1-S2

## Abbreviations

ELISA: enzyme-linked immunosorbent assay
LPS: lipopolysaccharide
miRNA: MicroRNA
NAD+: nicotinamide adenine dinucleotide
NAMPT: nicotinamide phosphoribosyltransferase
PBEF: Pre-B-cell colony-enhancing factor
qRT-PCR: reverse transcription-quantitative PCR
SIRT1: Sirtuin 1
UTR: untranslated region
