# Supplementary material for: MiR-96-5p alleviates inflammatory responses by targeting NAMPT and regulating the NF-κB pathway in neonatal sepsis
Source: Biosci Rep. 2020 Jul 3;40(7):BSR20201267. doi: 10.1042/BSR20201267 (PMC7335832; doi:10.1042/BSR20201267)
Supplement: Supplementary Figures S1-S2 [file BSR-2020-1267_supp.pdf]

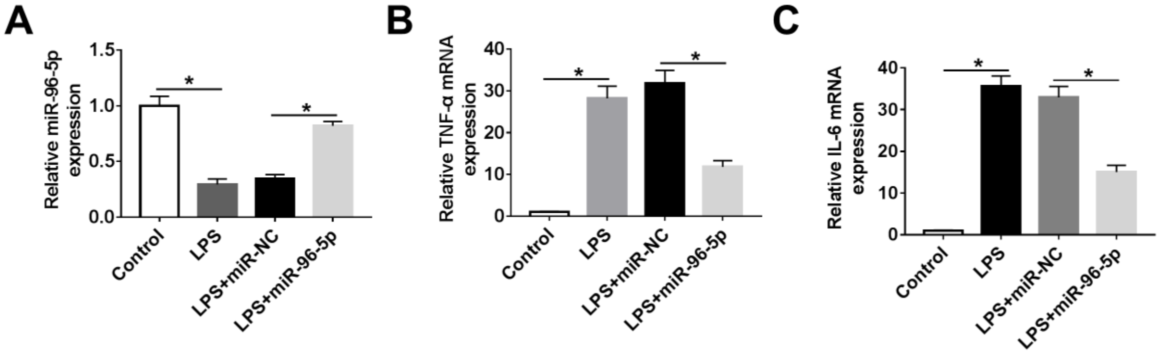

Fig. S1 miR-96-5p inhibited inflammatory response in monocytes from neonatal sepsis patients' blood samples. A-C, monocytes were treated with control, LPS, LPS + miR-NC or LPS + miR-96-5p mimic. **A,** The relative expression levels of miR-96-5p in cells were measured by qRT-PCR. **B and C,** the expression levels of TNF-α and IL-6 in cells were detected by qRT-PCR. *P<0.05.

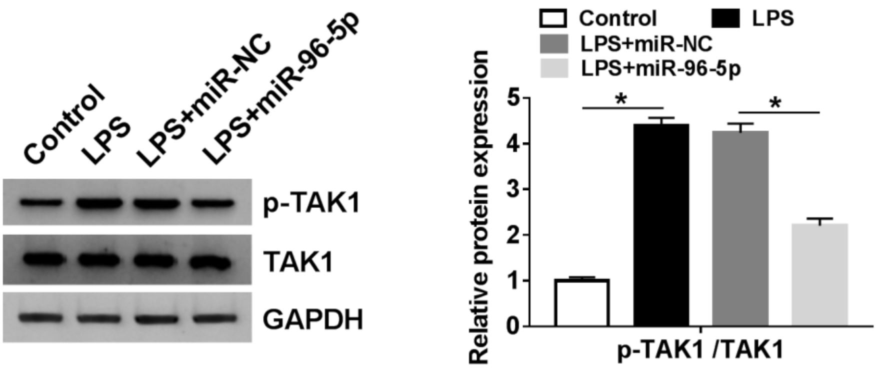

Fig. S2 MiR-96-5p inhibited TAK1 expression in RAW264.7 cells stimulated with LPS. The expression level of TAK1 in cells treated with LPS or LPS + miR-96-5p was detected by western blot. *P<0.05.
